# The complete mitochondrial genome sequence of *Eimeria leuckarti* (Eimeriidae, Coccidia, Apicomplexa) infecting domestic horses (*Equus ferus caballus*)

**DOI:** 10.1080/23802359.2021.1922318

**Published:** 2021-09-09

**Authors:** Evelin E. Rejman, Rosann Kehoe, John R. Barta

**Affiliations:** Department of Pathobiology, Ontario Veterinary College, University of Guelph, Guelph, Canada

**Keywords:** Coccidian parasite, Apicomplexa, mitochondrial genome, fragmented rRNA, equine host

## Abstract

The complete mitochondrial genome of *Eimeria leuckarti* (Eimeriidae, Coccidia, Apicomplexa) was obtained. This morphologically distinctive coccidium is considered to be the only valid *Eimeria* species of equids and it infects a range of both domestic and wild horses and their relatives. Despite the distinctive appearance of the oocysts of *E. leuckarti*, the mitochondrial genome organization and gene contents were comparable to other *Eimeria* spp. and related eimeriid coccidia infecting a range of mammals and birds. The greatly reduced 6242 bp genome is circular-mapping and contains three protein-coding genes (COI, COIII, CytB), 18 fragments encoding the large subunit rRNA (LSU), and 13 fragments encoding the small subunit (SSU) rRNA. No tRNA was encoded similar to other Apicomplexa. A Bayesian inference tree based on aligned CDS and rDNA fragments from *Eimeria leuckarti* and 34 other coccidia demonstrated that this mt genome has close phylogenetic affinities to *Eimeria* and *Isospora* species, and related eimeriid coccidia.

Coccidia are a diverse group of protozoan parasites that cause disease in a wide range of vertebrate hosts including equids (Tenter et al. 2002). As member of the family Eimeriidae Minchin 1903, *Eimeria* are host-specific, obligate intracellular protozoan parasites that have a complex life cycle with most species infecting the intestinal mucosa of the host (Lin et al. 2011). *Eimeria leuckarti* (Flesch 1883) Reichenow 1940 is the only coccidium known to infect the small intestine of equids (Barker and Remmler 1972) including domestic horses (*Equus ferus caballus*) (see Flesch 1883), donkeys (*Equus africanus asinus*) (see Reichenow 1940) and mules (*Equus africanus asinus*♂ × *Equus ferus caballus*♀) (see Postoli et al. 2010) as well as wild equids such *Equus hemionus*, *E*. *zebra* and *E*. *quagga boehmi* (see Tscherner 1978). There are no published molecular data from *Eimeria* species infecting equids. Although nuclear 18S rDNA is useful for classifying apicomplexan parasites (Morrison et al. 2004), nuclear rDNA cannot readily distinguish *Eimeria* spp. because of insufficient genetic diversity (Ogedengbe et al. 2011). In contrast, mitochondrial DNA (mtDNA) is a useful for both phylogenetic analyses and species delimitation (e.g. Liu et al. 2013).

*Eimeria* oocysts were isolated from feces of a young foal submitted to the Animal Health Laboratory, University of Guelph, Guelph, Ontario, Canada (43° 30′ N, 80° 12′ W) using standard flotation methods (Ryley et al., 1976). Mean dimensions of 50 oocysts (80 µm by 61 µm, shape index = 1.31) were found to be consistent with *Eimeria leuckarti* (see Battelli et al. 1995; Duszynski and Wilber 1997). A voucher phototype has been deposited with the Canadian Museum of Nature’s Invertebrate Collection (see https://nature.ca/en/contact-us) under accession number CMNPA 2021-0003; DNA extracted from the partially purified oocysts (CMNPA 2021-0003.1) and partially purified oocysts in 95% ethanol (CMNPA 2021-0003.2) have been deposited with the Canadian Museum of Nature’s National Biodiversity Cryobank of Canada. Unsporulated oocyst DNA was extracted as described (Ogedengbe et al. 2011) for PCR amplification of overlapping PCR amplicons spanning the mitochondrial genome using conserved primers (Ogedengbe et al. 2014). Chromatograms assembled within Geneious (V10.0.9, www.geneious.com, Biomatters Ltd., Auckland, New Zealand) provided a complete mitochondrial genome sequence that was annotated by comparison with the mt genome of *Eimeria innocua* mt genome (GenBank KR108296.1). The phylogenetic relationship of *E. leuckarti* to related coccidia was determined by extracting and concatenating all annotated regions (CDS and fragmented rDNA regions) prior to alignment. This alignment was partitioned to apply a codon-based nucleotide substitution model for the CDS partition and the GTR + I + G substitution model for the rDNA partition during phylogenetic analysis using Bayesian Inference (MrBayes Version 3.2.6.) (Huelsenbeck and Ronquist 2001).

The 6242 bp genome of *E. leuckarti* (GenBank MW354691; Rejman et al. 2021) is circular-mapping but its physical form remains uncertain; a related eimeriid coccidium, *Eimeria tenella*, possesses a linear concatemeric mt genome (Hikosaka et al. 2011). The mt genome of *Eimeria leuckarti* contains three protein-coding genes (CytB, COI and COIII) and numerous fragmented rDNAs (18 LSU and 13 SSU rDNA). Despite their noncontiguous nature, fragmented rDNAs are apparently transcribed (Feagin et al. 2012) and their conservation across diverse apicomplexan taxa suggests these fragmented rDNAs remain functional. The mitochondrial genome was strongly A + T biased (63.4%) especially within the CDS regions (67.4% CytB, 65.7% COI and 67.2% COIII).

The mt genome of *E. leuckarti* was related phylogenetically to various eimeriid coccidia (Figure 1). Unsurprisingly, the complement and organization of the mt genome of *E. leuckarti* are identical to mitochondrial genomes of other eimeriid coccidia including species of *Eimeria* (see Lin et al. 2011; Ogedengbe et al. 2014), *Isospora* (e.g. Hafeez and Barta 2017), *Caryospora* (e.g. Ogedengbe and Barta 2016) and *Cyclospora* (e.g. Cinar et al. 2015). Despite repeated attempts to amplify the nuclear 18S rDNA using a variety of PCR primers typically successful with apicomplexan taxa, no sequence data could be obtained from this nuclear locus. However, the mitochondrial genome and morphometrics are sufficient for differentiating *Eimeria leuckarti* from other eimeriid coccidia. The complete mitochondrial genome may provide a useful molecular target for diagnosing and tracing the spread of this coccidium among young equids.

**Figure 1.**
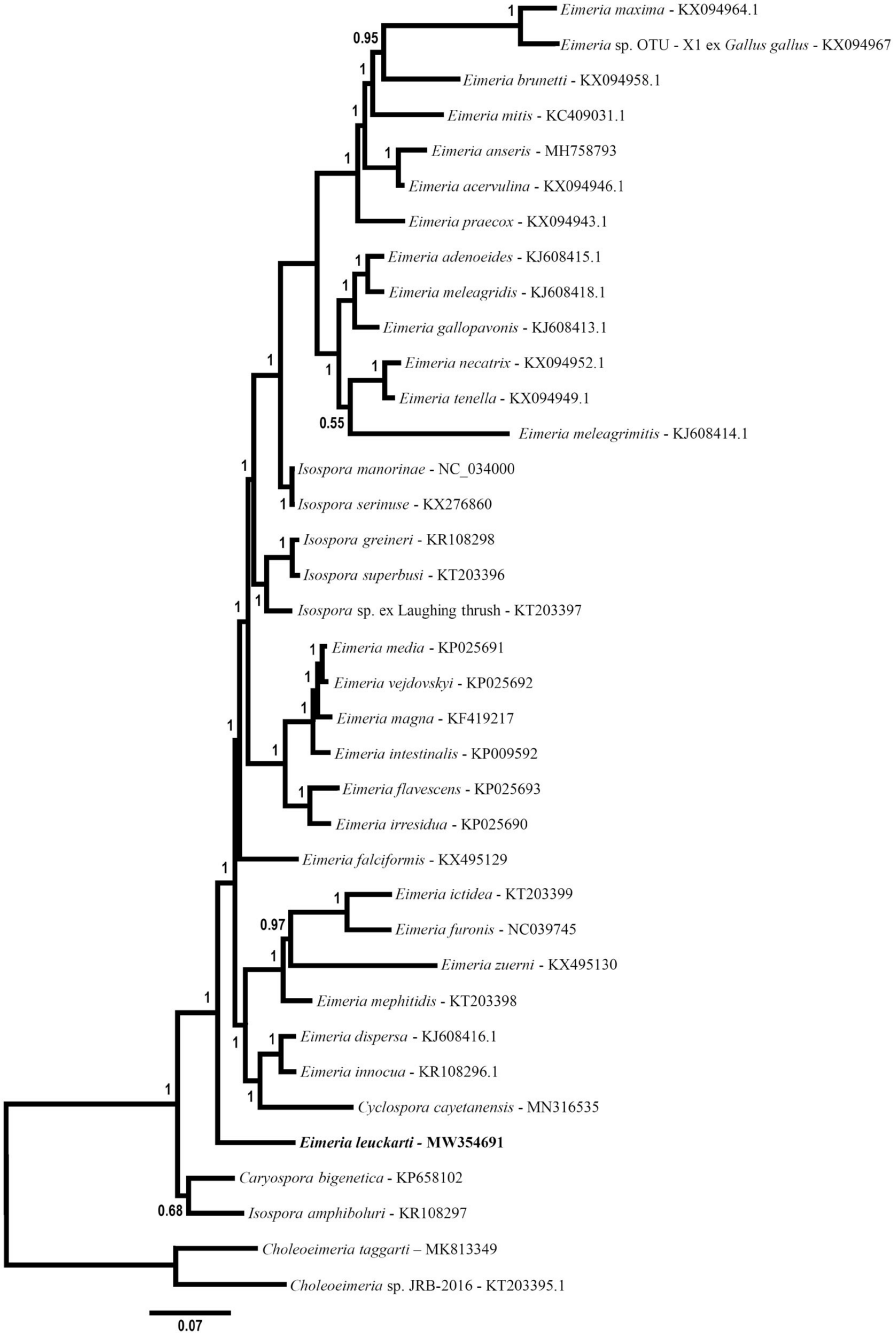
Phylogenetic relationship of the mt genome of *Eimeria leuckarti* among mitochondrial genomes of various eimeriid parasites inferred by Bayesian analysis of concatenated coding regions (COI, COIII, CytB, rDNA fragments). The resulting alignment was partitioned for Bayesian analysis as follows: CDS partition was analyzed using a codon-based nucleotide substitution model (i.e. Nucmodel = Codon; Code = MetMt) and the rDNA partition was analyzed simultaneously using a simpler general time reversible nucleotide substitution model with allowance for invariant characters and gamma distributed rate variation among sites (i.e. GTR + I + G; Nst = 6; Nucmodel = 4by4). Two *Choleoeimeria* species were used to root the tree. Horizontal distance is proportional to hypothesized evolutionary change and posterior probability support for each node is indicated.

## Data Availability

The genome sequence data are openly available in GenBank of NCBI at [https://www.ncbi.nlm.nih.gov] under accession no. MW354691, and additional data that support the findings of this study are available at Mendeley Data at http://dx.doi.org/10.17632/j6tjg4sdjf.2.
